# The Mutations and Clinical Variability in Maternally Inherited Diabetes and Deafness: An Analysis of 161 Patients

**DOI:** 10.3389/fendo.2021.728043

**Published:** 2021-11-25

**Authors:** Mengge Yang, Lusi Xu, Chunmei Xu, Yuying Cui, Shan Jiang, Jianjun Dong, Lin Liao

**Affiliations:** ^1^ Cheeloo College of Medicine, Shandong University, Department of Endocrinology and Metabology, Shandong Provincial Qianfoshan Hospital, Shandong Key Laboratory of Rheumatic Disease and Translational Medicine, Shandong Institute of Nephrology, Ji-nan, China; ^2^ Department of Endocrinology and Metabology, The First Affiliated Hospital of Shandong First Medical University & Shandong Provincial Qianfoshan Hospital, Ji-nan, China; ^3^ College of Traditional Chinese Medicine, Shandong University of Traditional Chinese Medicine, Ji-nan, China; ^4^ Division of Endocrinology, Department of Internal Medicine, Qilu Hospital of Shandong University, Ji-nan, China

**Keywords:** treatment, diagnosis, mitochondrial gene mutations, heteroplasmy, maternally inherited diabetes and deafness

## Abstract

**Aims:**

To investigate the clinical features and mitochondrial mutations for maternally inherited diabetes and deafness.

**Methods:**

PubMed, Embase, Medline, Web of Science, the China National Knowledge Infrastructure, and Wanfang were searched with the following search terms: “Maternally inherited diabetes and deafness” OR “MIDD” OR “Mitochondrial diabetes”. The mutations and clinical features were analyzed. Correlation between the heteroplasmy levels of the m.3243A>G mutation in the peripheral blood and age at the onset of diabetes was conducted by Spearman test. The significance level was set as p < 0.05. Statistical analysis was performed using the Statistical Package for the Social Sciences version 26 for Windows.

**Results:**

Totally 161 patients with 21 different mitochondrial mutations were enrolled. The most common mutation was the m.3243A>G mutation in 136 cases. Of 142 patients, 120 (84.51%) had family histories of diabetes or hearing loss. Hearing loss presented in 85.71% of the patients with mitochondrial mutations. Central nervous system diseases were found in 29.19%, myopathy in 22.98%, oculopathy in 23.60%, cardiac disease in 23.60%, and nephropathy in 13.66% of the patients. Forty-two of 101 (41.58%) patients were underweight. A significant negative correlation was found between the heteroplasmy levels of the m.3243A>G mutation in the peripheral blood and age at the onset of diabetes.

**Conclusions:**

The young onset of diabetes with low or normal BMI, maternal inheritance, and presence of impairments of multiple systems should prompt a genetic testing in order to differentiate MIDD from other types of diabetes earlier.

## Introduction

Diabetes mellitus is one of the most important chronic non-communicable disease, which has been a significant global public health problem. The incidence of monogenic diabetes mellitus has increased in recent years due to greater awareness and wider availability of genetic testing. Monogenic diabetes mellitus comprises a heterogeneous group of diabetes which are caused by a single gene defect (1). A subtype of monogenic diabetes associated with mutations in the mitochondrial DNA is referred to as maternally inherited diabetes and deafness (MIDD), which was first described in 1992 by Van den Ouveland et al. (2). The abnormality of glucose metabolism in MIDD is associated with a gradual decrease in insulin secretion due to reduced ATP production in pancreatic β-cells with abnormal mitochondria (3). The diagnosis and differentiation of MIDD from type 1 and type 2 diabetes are important in view of the implications for treatment and prognosis as well as for identification of family members at risk of diabetes (2). However, the clinical features of MIDD are variable, and MIDD is frequently misdiagnosed as other types of diabetes. Thus, it is extremely essential to recognize MIDD among the diabetic patients. It is a pity that there is no systematic summary about the disease up to now. Our study summarized the clinical features and mutations in reported MIDD to help the doctors better diagnose and manage these patients.

## Subjects and Methods

PubMed, Embase, Medline, Web of Science, the China National Knowledge Infrastructure (CNKI), and Wanfang were searched from the date of their inception to February 10, 2021, without language restrictions. The key words used were “Maternally inherited diabetes and deafness” OR “MIDD” OR “Maternally inherited diabetes” OR “Mitochondrial diabetes.” Eligible studies met the following criteria: (1) Mitochondrial mutations were confirmed by genetic testing, and detection of the mutation was based on polymerase chain reaction (PCR) amplification. (2) The mutation sites were described. (3) The main clinical data of patients were described. The flow chart (**Supplementary Data Figure S1**) showed the reasons for identification of eligible studies.

From the eligible studies, the following data were extracted: (1) country, (2) gender, (3) age at onset of diabetes and hearing loss, (4) family history, (5) therapies, (6) BMI, (7) fasting C-peptide, (8) mutation sites, (9) antibodies, (10) the heteroplasmy levels in tissues.

### Statistical Analyses

Correlation between the heteroplasmy levels of the m.3243A>G mutation in the peripheral blood and age at the onset of diabetes was conducted by Spearman test. The significance level was set as p < 0.05. Statistical analysis was performed using the Statistical Package for the Social Sciences version 26 for Windows (SPSS).

## Results

### Epidemiological Characteristics and Gene Mutations

**Figure 1:**
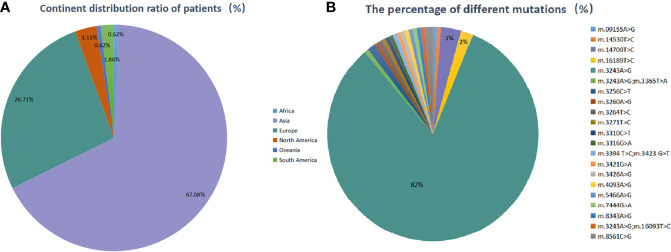
**(A)** Geographical country distribution ratio among the patients (%), **(B)** the percentage of different mutation sites (%).

One hundred and sixteen studies including 161 patients were eligible with the aforementioned search terms. Among them, Asian cases accounted for the largest part (108 cases, 67.08%), followed by European (43 cases, 26.71%), North American (5 cases, 3.11%), South American (3 cases, 1.86%), African and Oceania (1 case, 0.62%, respectively). Among the Asian, cases from China, Japan, India, Iran, and Korea accounted for 39.75, 25.47, 0.62, 0.62, and 0.62%, respectively. Totally 21 different mtDNA mutations were identified. Most of the patients (158/161, 98.14%) had one mutation, and three (3/161, 1.86%) had two mutations. The most common mutation was the m.3243A>G mutation, which accounted for 84.47% of the cases, while other mutations were quite rare. The detailed information of enrolled countries and individuals are described in **Table S1** (**Supplementary Materials**) and **Figure 1**.

### Clinical Characteristics

**Figure 2:**
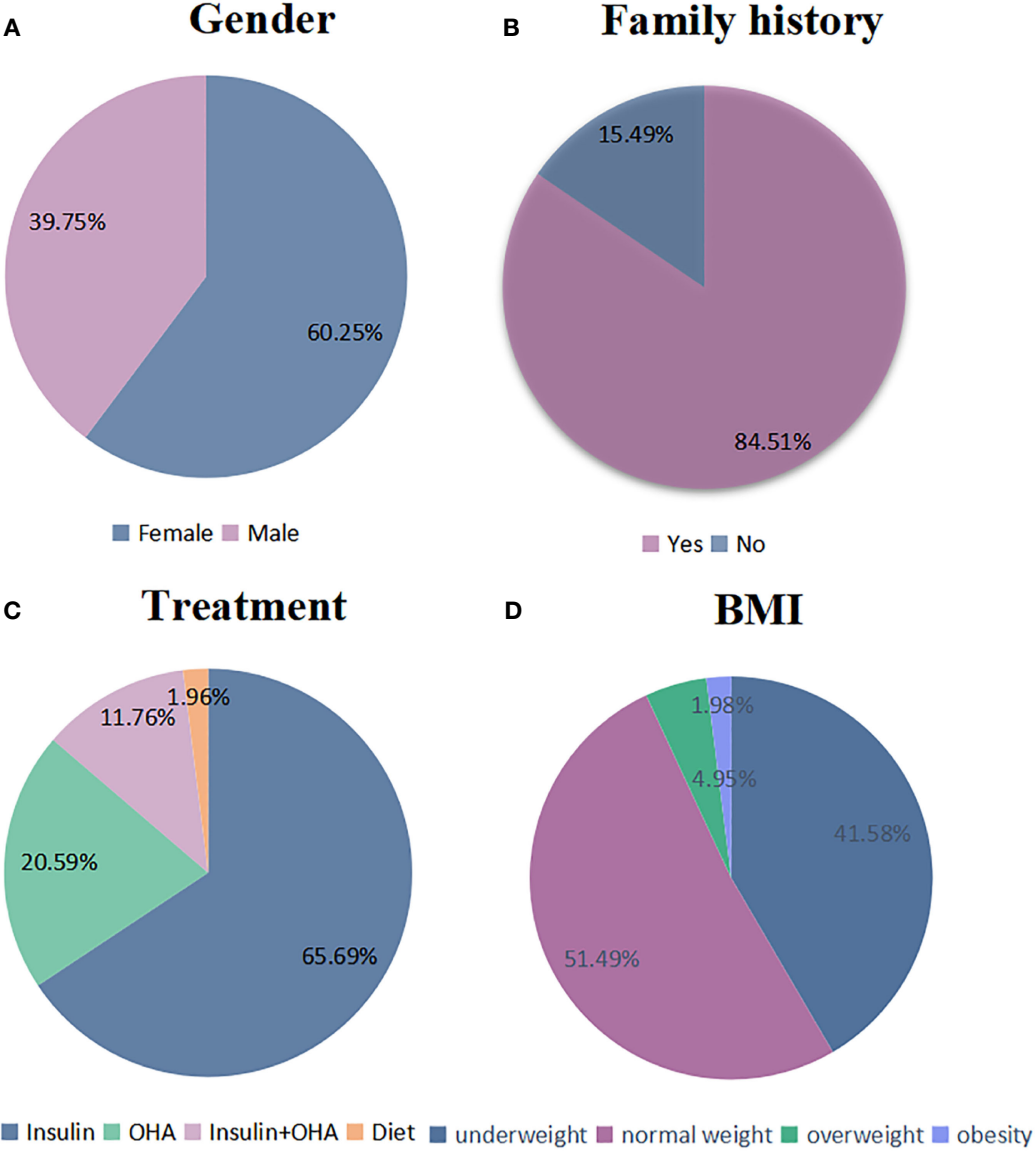
Clinical characteristics of patients with mitochondrial mutations. **(A–D)** The proportion of several clinical characteristics in enrolled patients: **(A)** gender (N: 161), **(B)** family histories (N: 142), **(C)** treatment (N: 102), and **(D)** BMI (N: 101).

**Figure 3:**
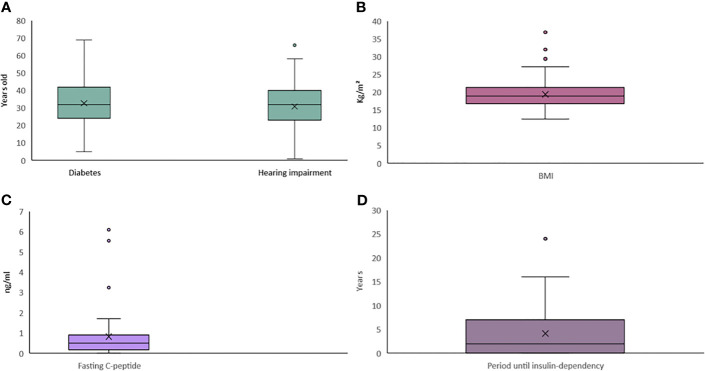
Whisker plot for continuous clinical data of patients with mitochondrial mutations. **(A–D)** Continuous data for the variables of **(A)** Age at onset, **(B)** BMI, **(C)** Fasting C peptide, and **(D)** Period until insulin dependency.

The clinical data at diagnosis of the patients are shown in **Table S2** (**Supplementary Materials**) and **Figures 2** and **3**. Among the 161 patients, 60.25% (97/161) were female and 39.75% (64/161) were male. Family histories were mentioned in 142 patients, and among them, 59.86% (85/142) had family histories of both diabetes and hearing loss; 21.83% (31/142) had family history of diabetes; 2.82% (4/142) had family history of hearing loss. Mitochondrial mutations were detected in 3.52% (5/142) of patients’ relatives who had no hyperglycemia or hearing loss. The body-mass-index (BMI) at diagnosis were available in 101 patients. Their BMI ranged from 12.5 to 36.89 kg/m² (mean 19.41 kg/m²). The group of underweight (<18.5 kg/m^2^), normal weight, overweight (25~29.9 kg/m^2^), or obese (≥30 kg/m^2^) accounted for 41.58, 51.49, and 6.93%, respectively, according to WHO standard.

Fasting C-peptide data were available in 42 patients, with a mean of 0.83 ng/ml, lower than the normal range (1.1~4.4 ng/ml). Diabetes antibodies were reported in 50 patients, and among them, only five (10.00%) patients had positive results (one case had GADA, two cases had IAA, and two cases had ICA).

The clinical manifestations of MIDD patients are shown in **Table S3** (**Supplementary Materials**). Hearing impairment presented in 138 (85.71%) patients. Central nervous system diseases were found in 47 (29.19%) patients, including encephalatrophy (11.18%), cerebellar ataxia (6.21%), basal ganglia calcification (2.48%), migraine (4.97%), and cerebral infarction (5.59%). Peripheral neuropathy was found in 28 (17.39%) patients. Myopathy was presented in 37 (22.98%) patients manifesting muscle weakness (14.91%), myophagism (4.97%), ptosis (2.48%). Ragged red fibers on muscle biopsy were seen in 26 (16.15%) patients. Oculopathy was observed in 38 (23.60%) patients, and the prevalence of macular degeneration (9.32%) was higher than proliferative retinopathy (5.59%). Cardiac disease was present in 38 (23.60%) patients, and 19 (11.80%) patients had a manifestation of ventricular hypertrophy and 15 (9.32%) patients had a manifestation of arrhythmia. Nephropathy, defined by the presence of albuminuria and/or impairment in renal function, was found in 22 (13.66%) patients. Gastrointestinal symptoms were observed in 9 (5.95%) patients. In addition to diabetes, other endocrine disorders involving hypogonadism (1.86%) and osteoporosis (2.48%) were observed.

### Treatment

The treatments of 102 patients were provided in the original articles. Seventy-nine patients (79/102, 77.45%) injected insulin, and 23 patients (23/102, 22.55%) did not. Among them, 67 patients (67/102, 65.69%) received insulin without oral hypoglycemic agent. Twenty-two patients (21/102, 20.59%) received oral hypoglycemic agents (OHA) without insulin, and 12 (12/102, 11.76%) received both insulin and OHA. Two patients (2/102, 1.96%) underwent diet therapy only. Among the patients who received insulin, 37.18% (29/78) initiated once diabetes was diagnosed. The mean time from the diagnosis of diabetes to insulin therapy was 4.15 years. Onset of the diabetes usually occurred at an early age with a mean age of 32.79 years, and the mean age of onset of hearing impairment was 30.84 years.

### Heteroplasmy Levels

**Figure 4:**
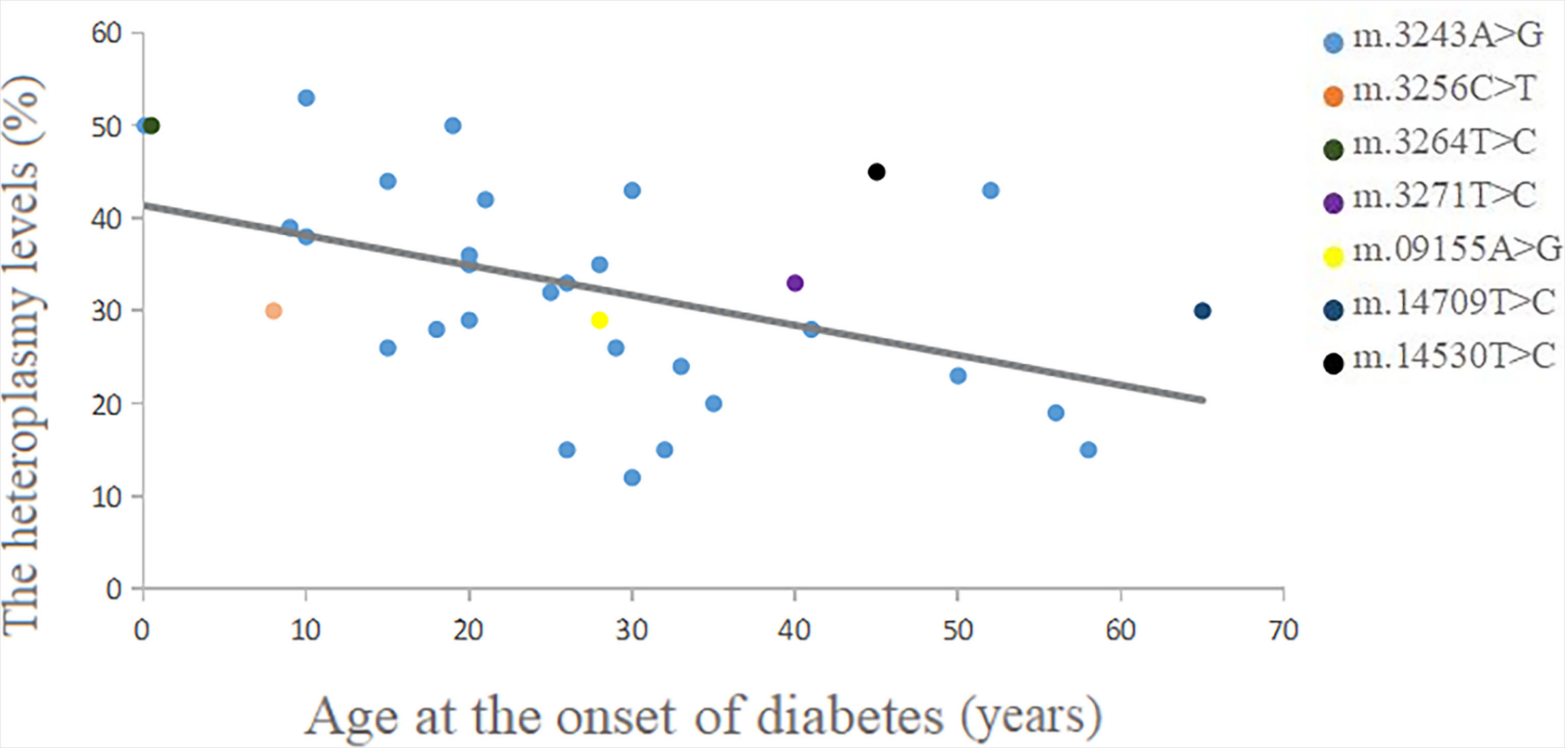
Correlation between blood heteroplasmy levels and age at the onset of diabetes.

The heteroplasmy levels of the m.3243A>G mutation in different tissues are shown in **Table 1**. The blood heteroplasmy levels in 27 patients ranged from 0.102 to 58%, with a mean value of 26.97%. The mean heteroplasmy levels in urothelium, muscle, hair follicle, buccal mucosa, nail, and myocardium were 58.13, 49.53, 41.35, 48.25, 32.00, and 67.50% respectively. We used different colors representing different mutations in the scattergram, which showed a negative correlation between blood heteroplasmy levels and age at the onset of diabetes (**Figure 4**). A significant negative correlation was found between the blood mutation levels of the m.3243A>G mutation and age at the onset of diabetes (P<0.01) (**Table 2**).

**Table 1:** The heteroplasmy levels of the m.3243A>G mutation in different tissues.

Tissue	N	Heteroplasmy levels (%)		
		Min	Max	Mean
Peripheral blood leukocyte	27	0.102	58	26.97
Urothelium	3	31.4	76	58.13
Muscle	7	0.024	90	49.53
Buccal mucosa	4	40	58	48.25
Hair follicle	2	12.7	70	41.35
Nail	1	32	32	32.00
Myocardium	2	60	75	67.50

N, number of patients.

**Table 2:** Correlation between blood heteroplasmy levels of the m.3243A>G mutation and age at the onset of diabetes.

Age at onset	The heteroplasmy levels in peripheral blood	blood
	r	p
Diabetes	−0.612	0.001^**^

**p < 0.01

## Discussion

Since MIDD was first described, there have been some reports about this disease. However, most studies about MIDD involved only a small series of patients. Unlike previous studies, we summarized the clinical features and mutations of 161 patients with mitochondrial mutations. Our study demonstrated the MIDD patients with most mutations had the following clinical characteristics: (1) high incidence of progressive neurosensory deafness (85.71%); (2) early onset of diabetes and deafness; (3) high incidence of maternal inheritance (84.51%); (4) thin and short stature (41.58%); (5) absence of diabetes antibodies (90.00%); (6) progressive insulin secretory defect.

But not all patients with mitochondrial mutations met the above characteristics. The m.3426 A>G and m.16189T>C mutation were considered as susceptibility factors for insulin resistance and T2DM, which were also maternal inherited (4–6). The patients with m.3426 A>G mutation did not have deafness and had late onset age of diabetes (mean 42.14 years) with the BMI above the normal range (mean 27.14 kg/m²) and usually had no complications (4). In addition, the laboratory characteristics of patients with m.3426A>G mutation was that they had normal levels of fasting C-peptide and usually took oral hypoglycemic agents for treatment (4, 6). The patients with m.16189T>C mutation were also usually overweight (mean BMI 28.12 kg/m²) and had high HOMA-IR (5). The reason for the above differences is not clear yet. Previous studies have demonstrated the m.3243A>G mutation is associated with both insulin deficiency and reduced insulin sensitivity in patients with MIDD (7). Sequential follow-up of patients with the 3243 mutation documented decreased sensitivity to insulin prior to the development of insulin deficiency (7). A presumable supposition is that reduced sensitivity to insulin could be a consequence of minor mitochondrial dysfunction, and deficiency only occurred with a severe defect. The mutations 3426A>G and 16189T>C have less impact on mitochondrial function than 3243A>G.

MtDNA is passed from the mother to offspring; therefore, it is generally accepted mitochondrial diabetes is also maternally inherited (8, 9). However, in our study, 22 patients were sporadic cases without family histories of diabetes and hearing loss. Among the 22 patients, mitochondrial mutations were detected in five patients’ relatives who had no hyperglycemia or hearing loss. One possible reason for this seems to be that mitochondrial inheritance is characterized by heteroplasmy and threshold (10). The percentage of affected mitochondria is variable among different tissues and among cells within a given tissue, which is called heteroplasmy (11). Typically, the carrier exhibits clinical phenotypes when the mutant mtDNA reaches more than 60%, which is the threshold (12). The other reason is that the patient was the propositus and had the first mutation in his family (13).

In addition to diabetes mellitus and hearing loss, other clinical phenotypes may present differently since there are variabilities in heteroplasmy in different tissues (14, 15). Mitochondria are presented in every cell of the human body except red blood cells, so the organs depending on energy metabolism are subjected to be affected in patients with MIDD, such as central nervous system, retina, skeletal muscle, heart, kidney, and endocrine. We found some complications involving other systems are characteristic, which might help to identify MIDD from other types of diabetes.

The prevalence of macular dystrophy (9.32%) was higher than proliferative retinopathy (5.59%), which was consistent with a previous study (16). Macular pattern dystrophy might protect against the development of diabetic retinopathy through a reduction in retinal metabolism and a decrease in oxygen consumption in the retina (17). Macular dystrophy is thought be used as a marker to select the MIDD patients (17). This typical retinal dystrophy should raise suspicion as to the diagnosis for MIDD. Therefore, it is of clinical importance for diabetes to perform specific examinations by a trained ophthalmologist.

Our results also suggested a high frequency of myopathy, especially muscle weakness and myophagism. Skeletal muscle requires a great deal of ATP to function, and when patients increase the demand on the muscles, the dysfunctional mitochondria are unable to meet the demand and develop muscular symptoms (18). Further, ragged red fibers on muscle biopsy are typically seen in MIDD, which indicate damaged muscles and are thought to be pathognomonic of mitochondrial disease (19). Muscle biopsy may show ragged-red fibers that are characteristic of mitochondrial disorders and contribute to the diagnosis of MIDD (20).

In the UK MRC Mitochondrial Disease Patient Cohort Study involving 129 patients with the m.3243A>G mutation, the patients were associated with other clinical syndromes involving the nervous and muscular systems including mitochondrial encephalopathy lactic acidosis and stroke-like episodes (MELAS) and chronic progressive external ophthalmoplegia (CPEO). Thirty percent had MIDD, 6% MELAS/MIDD, and 5% MIDD/CPEO overlap syndromes (21). Our study also showed high prevalence of central nervous system involvement (29.19%) and myopathy (22.98%) in MIDD patients. Brain and skeletal muscle are susceptible to be affected due to a great demand for ATP (3). Basal ganglia high signal lesions (2.48%) or encephalatrophy (11.18%) on the brain computerized tomography (CT) are specific manifestations, which might have implications for the diagnosis.

Cardiac abnormalities in MIDD mainly manifested as cardiomyopathy or arrhythmias. The decrease in ATP leads to a decrease in contractility and subsequently results in decreased stroke volume. The increase in end diastolic volume and pressure in the left ventricle causes further left ventricular remodeling, most often, left ventricular hypertrophy (22). Cardiac conductance disorders might occur by a rearrangement of the cardiac conductance system induced by the mitochondrial dysfunction. Common cardiac conduction abnormalities seen in MIDD include Wolff Parkinson White syndrome, frequent ventricular extrasystoles, and atrial fibrillation (20, 22). Cardiac conductance disorders might contribute to sudden deaths; therefore, patients diagnosed with MIDD should undergo a comprehensive cardiac examination to avoid adverse outcomes (15).

Although the prevalence of nephropathy (13.66%) and gastrointestinal symptoms (5.59%) was relatively lower, it was not insignificant. Renal biopsy of MIDD often shows focal glomerulosclerosis (FSGS), not typical diabetic nephropathy, and is the most prevalent finding (23). The main gastrointestinal symptoms were usually mild and presented as constipation, diarrhea, or succession of both. Intestinal pseudo-obstruction is a rare but serious complication of mitochondrial disease of alimentary localization with a mortality rate of approximately 50% (24). In addition, there have also been rare reports of pancreatitis, which may be related to MIDD (25, 26). Apart from diabetes, the most common endocrine manifestation of MIDD was short stature. Hypothyroidism and hypogonadism were also observed in MIDD (27–29). For MIDD patients, in addition to glycemic control, other endocrine function should be cared.

In clinical practice, testing for mutations in mitochondrial DNA is routinely done in DNA isolated from blood; however, the level of mutation may be low and even undetectable in blood. Our study indicated that mutational loads of m.3243A>G varied widely among different tissues. The mean mutation levels of m.3243A>G in urothelium, muscle, hair follicle, buccal mucosa, nail, and myocardium were 58.13, 49.53, 41.35, 48.25, 32.00, and 67.50%. DNA from urothelium, myocardium, and buccal mucosa had the higher while blood had the lowest proportion of mutant genomes. These results are consistent with a previous research (20). Each time a somatic cell undergoes mitosis, mtDNA will be randomly distributed to the progeny cells along with mitochondria. Therefore, the mutation load of mtDNA in the tissue will change with the division of the tissue cells (30, 31). The above results indicate that buccal mucosa and urinary sediment are tissues of choice for the diagnosis of mtDNA mutations, as they are easy to obtain and their mutation loads are greater than blood.

Five patients were positive for diabetes antibodies. The patient with GADA positive was diagnosed as MIDD combined with latent autoimmune diabetes in adults (LADA) (32). The two patients with ICA positive were diagnosed as MIDD. The original literatures considered that metabolic damage to beta cells could possibly trigger an autoimmune response and ICA might be a secondary phenomenon in mitochondrial diseases (33, 34). As for two patients with IAA positive, one was diagnosed as MIDD (32). The other patient repeated antibody tests, and the results were negative for all antibodies in the first test and positive for IAA in the second test. Since the patient had high HOMA-IR, the patient was diagnosed as T2DM combined with mitochondrial gene mutation (35). Actually, the three diabetes antibodies all have false-positive rates, which can be positive in some patients without diabetes, and some drugs such as methimazole could result in positive antibodies (36, 37).

We investigated the correlation between the heteroplasmy levels in the peripheral blood and age at the onset of diabetes. The scattergram showed an overall trend that the age at onset of diabetes was negatively correlated with the heteroplasmies in the peripheral blood. Especially the age at onset of m.3243A>G was significantly negatively correlated with the heteroplasmies in the peripheral blood, which was consistent with previous studies (38). The heteroplasmy load of the m.3243A>G mutation declines with age in blood leukocytes at a mean rate of 1.4% per year (39). Moreover, as the patient’s age increased, all tissue showed a declining proportion of mutant mtDNA (40), which might indicate that early genetic testing is easier to detect mutations, and the positive rate of detection decreases with age. And one previous study suggested age-adjusted blood m.3243A>G mutation load might be an indicator of disease burden (41).

For MIDD patients, the clinical management is also a complicated issue to be discussed. Our study showed that most of the MIDD patients (79/102,77.45%) received insulin therapy, 37.18% (29/78) initiated insulin once diabetes was diagnosed, and less than one-fourth (24/102,23.53%) did not use insulin. The mean time from the diagnosis of diabetes to insulin therapy was 4.15 years. Our results might indicate that only minority of MIDD patients did not need insulin injection as they were diagnosed. As islet function declines, insulin is usually required. There is a research suggesting a cocktail therapy combining CoQ10, lipoic acid, L-carnitine, and thiamine (42). CoQ10 is an antioxidant and mitochondrial cofactor that plays an important role in the mitochondrial respiratory chain, which may enhance insulin secretion, slow hearing loss, improve symptoms of myopathy and congestive heart failure in the setting of mitochondrial disease (43–45). Some drugs should be avoided, such as metformin as it might increase the risk of lactic acidosis (46). Additionally, some antibiotics such as tetracycline (47) and chloramphenicol (48), antiepileptic drugs such as sodium valproate phenytoin and phenobarbital (49), antiretroviral drugs (50), and statins (51) should be taken carefully due to their mitochondrial toxicity, which may exacerbate mitochondrial damage. For hearing impairment, cochlear implantation could improve listening in patients of all ages, provided there are intact neural components to function (52).

Our study has several limitations. First, to analyze the characteristics of patients with MIDD, studies with insufficient information were excluded, so that we were unable to analyze the characteristics of some rare mutations. Second, this study might have selective bias, because typical or more severe patients tend to be reported. Additionally, the mechanism of different mutations leading to various clinical features still remains unclear. Further studies are needed for the purpose of explaining the more precise molecular mechanism of MIDD.

## Conclusions

In summary, our study shows that MIDD is a heterogeneous disease which is various in clinical features and treatment. The age of onset of diabetes is negatively correlated with the heteroplasmies in the peripheral blood. These patients who present with low body mass index, young onset of diabetes but without positive antibodies, multiple systems involved especially hearing loss should prompt an investigation for MIDD and genetic counseling as soon as possible.

## Author Contributions

MY contributed to the conception and design of the study. CX, YC, and LX performed the literature search and study selection. LX performed the data extraction. MY and SJ performed the statistical analyses. MY, LL, SJ and JD drafted the manuscript and revised it critically. SJ polished the draft. All authors gave final approval for submitted manuscript content and agreed to be accountable for all aspects of the work in ensuring that questions related to the accuracy or integrity of any part of the work are appropriately investigated and resolved.

## Funding

This work was funded by the National Natural Science Foundation of China (81770822).

## Conflict of Interest

The authors declare that the research was conducted in the absence of any commercial or financial relationships that could be construed as a potential conflict of interest.

## Publisher’s Note

All claims expressed in this article are solely those of the authors and do not necessarily represent those of their affiliated organizations, or those of the publisher, the editors and the reviewers. Any product that may be evaluated in this article, or claim that may be made by its manufacturer, is not guaranteed or endorsed by the publisher.

## Abbreviations

MIDD, maternally inherited diabetes and deafness; mtDNA, mitochondrial DNA; tRNA, transfer RNA; PCR, polymerase chain reaction. GADA, glutamic acid decarboxylase antibody; IAA, insulin autoantibody; ICA, islet cell antibody; OHA, oral hypoglycemic agents; MELAS, mitochondrial encephalopathy lactic acidosis and stroke like episodes; CPEO, chronic progressive external ophthalmoplegia; FSGS, focal glomerulosclerosis.
