# Supplementary material for: The Mutations and Clinical Variability in Maternally Inherited Diabetes and Deafness: An Analysis of 161 Patients
Source: Front Endocrinol (Lausanne). 2021 Nov 25;12:728043. doi: 10.3389/fendo.2021.728043 (PMC8654930; doi:10.3389/fendo.2021.728043)
Supplement: Supplementary file 1 [file DataSheet_1.pdf]

Figure S1 Literature review inclusion process.

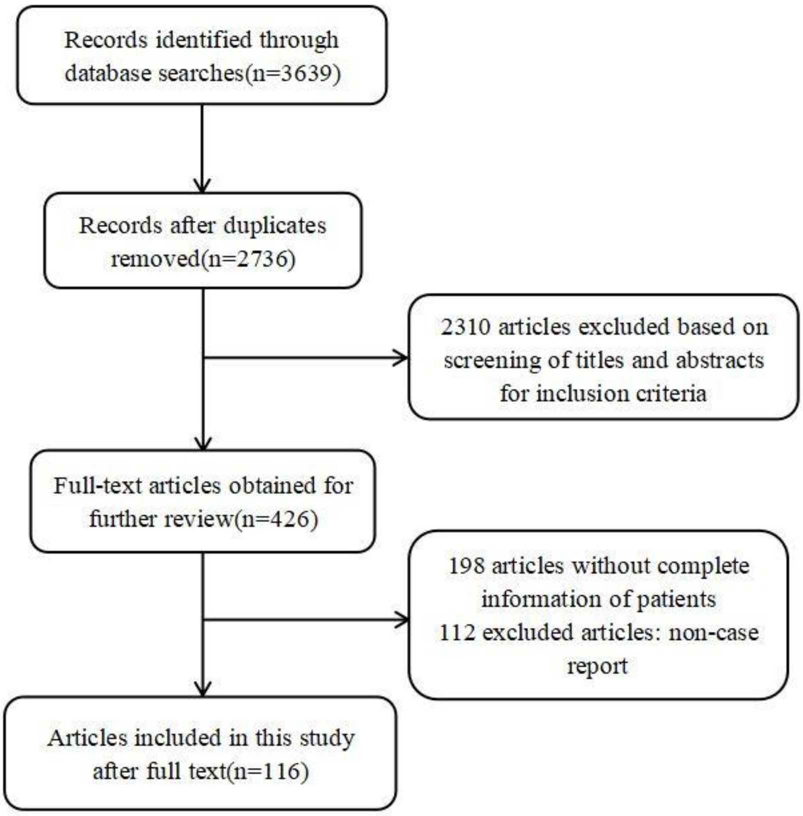

Table S1 The detailed information of MIDD studies.

Continent	Enrolled articles	Mutation site	Patients (Percentage%)
Asia	72	m.3243A>G	90 (55.90)
		m.16189T>C	3 (1.86)
		m.14709T>C	1 (0.62)
		m.3243A>G;m.3365T>A	1 (0.62)
		m.3256C>T	1 (0.62)
		m.3264T>C	1 (0.62)
		m.3271T>C	1 (0.62)
		m.3310C>T	1 (0.62)
		m.3316G>A	1 (0.62)
		m.3394 T>C;m.3423 G>T	1 (0.62)
		m.3421G>A	1 (0.62)
		m.3426A>G	1 (0.62)
		m.4093A>G	1 (0.62)
		m.5466A>G	1 (0.62)
		m.7444G>A	1 (0.62)
		m.8343A>G	1 (0.62)
		m.3243A>G; m.16093T>C	1 (0.62)
Europe	35	m.3243A>G	35 (21.74)
		m.14709T>C	3 (1.86)
		m.8561C>G	2 (1.24)
		m.3260A>G	1 (0.62)
		m.14530T>C	1 (0.62)
		m.09155A>G	1 (0.62)
North America	4	m.3243A>G	3 (1.86)
		m.14709T>C	1 (0.62)
		m.3256C>T	1 (0.62)
Africa	1	m.3243A>G	1 (0.62)
Oceania	1	m.3243A>G	1 (0.62)
South America	3	m.3243A>G	3 (1.86)
Total	116		161 (100.00)

Table S2 Clinical data of MIDD patients.

Subjects	No. of patients	Mean±SD
Age at onset of diabietes (yr)	159	32.79±12.74
Age at onset of hearing impairment (yr)	85	30.84±13.10
BMI (Kg/m^2)	101	19.41±3.85
Fasting C-peptide (ng/ml)	42	0.83±1.28
Blood lactic acid (mmol/l)	21	3.03±1.03
Period until insulin-dependency (yr)	67	4.15±5.24

Table S3 Symptoms and signs of MIDD patients.

Symtoms & signs	Enrolled articles	Cases	Prevalence(%)
**Hearing loss**	99	138	85.71
**Central nervous system**	42	47	29.19
Encephalatrophy		18	11.18
Cerebellar ataxia		10	6.21
Basal ganglia calcification		4	2.48
Neuropsychological deficits		7	4.35
Migraine		8	4.97
Cerebral infarction		9	5.59
**Myopathy**	35	37	22.98
Muscle weakness		24	14.91
Myophagism		8	4.97
Ptosis		4	2.48
Ragged red fibers		26	16.15
**Polyneuropathy**	17	28	17.39
**Eyes**	36	38	23.60
Macular degeneration		15	9.32
Proliferative retinopathy		9	5.59
**Heart**	31	38	23.60
Ventricular hypertrophy		19	11.80
Arrhythmia		15	9.32
**Endocrine system**	116	161	100.00
Diabetes		161	100.00
Hypogonadism		3	1.86
osteoporosis		4	2.48
**Nephropathy**	19	22	13.66
**Gastrointestinal symtoms**	9	9	5.59
